# Reading and math skills development among Finnish primary school children before and after COVID-19 school closure

**DOI:** 10.1007/s11145-022-10358-3

**Published:** 2022-09-27

**Authors:** Marja-Kristiina Lerkkanen, Eija Pakarinen, Jenni Salminen, Minna Torppa

**Affiliations:** Department of Teacher Education, University of Jyväskylä, P.O. Box 35, 40014 Jyväskylä, Finland

**Keywords:** Reading, Mathematics, Task avoidance, Homework, COVID-19

## Abstract

This study quantified the possible learning losses in reading and math skills among a sample of Finnish Grade 3 children (*n* = 198) who spent 8 weeks in distance learning during the first wave of the COVID-19 pandemic in spring 2020. We compared their reading and math skill development trajectories across Grades 1, 2, and 4 to a pre-COVID sample (*N* = 378). We also examined if gender, parental education, maternal homework involvement, and child’s task-avoidant behavior predict children’s academic skills at Grade 4 differently in the pre-COVID sample compared with the COVID sample. Children’s reading and math skills were tested, mothers reported their education and homework involvement, and teachers rated children’s task-avoidant behavior. The results showed, on average, lower reading skills in the COVID sample than in the pre-COVID sample but there were no differences in math skills. Although the COVID sample had lower levels in reading, their developmental trajectories in reading and math skills were not different from the pre-COVID sample before the pandemic in Grades 1 and 2. From Grade 2 to 4, however, the development was slower in reading fluency and comprehension in the COVID sample, but not in math. The predictors of change from Grade 2 to 4 in reading and math skills were not different in the samples. The results showed that the development of reading skills in particular may have been affected by the COVID-19 pandemic.

## Introduction

The COVID-19 pandemic has challenged education globally. Because of the pandemic, schools have been closed for several weeks or even months, relying on online distance learning, which has decreased learning gains and increased stress levels of students, teachers, and parents. In addition, quarantines and other changes in school practices have affected children’s everyday schooling since the pandemic began. The pandemic has also highlighted the increasing polarization in education due to a number of factors. First, there was a wide variety of regional solutions and restrictions as a result of COVID-19 (UNESCO, 2022). Second, there have frequently been challenges in digital infrastructure and technical capacities in schools, such as inadequate learning environments at home and unequally distributed resources in home and school contexts regarding access to facilities and information and communication technology (ICT) devices for distance learning (Huber & Helm, 2020). Third, parents had differential resources for coaching their children during school closure (Van Bakel et al., 2022). Fourth, it has been found that most students have spent less time on learning and put less effort into learning tasks (Grewenig et al., 2021; Huber & Helm, 2020). A tendency for the most severe learning losses in reading and math due to the pandemic has been reported, especially among low-achieving students (Schult et al., 2022). Therefore, there is serious concern that the school closures during the COVID-19 pandemic have produced substantial losses in academic outcomes and that the risk of dropout from education, especially among the most vulnerable children, has been intensified. However, there is currently rather limited evidence on the possible learning losses from different educational contexts, and the findings are contradictory (Hammerstein et al., 2021). Still, little is known about the role of the COVID-19 pandemic and the related school closure and home environment in predicting the developmental trajectories in reading and mathematics for primary school students.

In Finland, schools were closed for 8 weeks in March 2020, and instruction switched to remote teaching. To help mitigate the effect of school closures, there were policy recommendations for schools to immediately organize distance learning. Since the advent of the remote teaching period, there have been various reports that remote teaching practices have influenced teachers’ well-being (Pöysä et al., 2022), students’ well-being (Hämeenaho & Sainio, 2021), and their parents’ well-being (Sorkkila & Aunola, 2021). However, evidence on learning losses during the pandemic is scant in Finland. This study quantified the possible learning losses in reading and math among a sample of Finnish children who were in Grade 3 when the pandemic began in spring 2020 and who spent 8 weeks in distance learning at home. We compared their reading and math test scores across Grades 1, 2, and 4 with the pre-pandemic assessments of a pre-COVID sample from 2008 to 2011. In addition, we examined whether the COVID and the pre-COVID samples differed as a function of gender, parental education, maternal homework involvement, and children’s task-avoidance behavior. The aim of the present study was to provide empirical evidence on the possible learning loss affected by school closure during the COVID-19 pandemic in Finland, where educational level has been repeatedly reported to be one of the highest among PISA surveys (OECD, 2019).

### The development of reading and math skills during the pandemic

The rapid move to remote online teaching gave students, teachers, and parents little time to prepare children for distance learning. Empirical evidence on the impact of COVID-19-related school closures in primary and secondary education has begun to emerge only recently. Decline in student achievement, specifically in younger students, students with a migration background, and students from less-educated family backgrounds has been reported (Maldonado & De Witte, 2021). However, the systematic review by Hammerstein et al. (2021) reported mixed findings across studies, with effects ranging from − 0.37 to + 0.25 *SD.*

Most studies have estimated average learning losses in reading and math (e.g., Engzell et al., 2021; Georgiou, 2021; Kuhfeld et al., 2020; Maldonado & De Witte, 2021; Schult et al., 2021). For example, a study of fifth graders’ samples before and during the pandemic (each sample *n* > 80,000) in German schools reported that student achievement was slightly lower in 2020 compared with the student achievement for the previous three years both in reading comprehension and in mathematics (Schult et al., 2021). In Schult et al.’s (2021) study, family socio-economic status (SES) and migration background played a minor role in learning loss, although lower SES was significantly associated with larger learning loss in mathematics. In the Netherlands, the 8-week school closure in spring 2020 was associated with learning losses in reading, spelling, and math in Grades 4 to 7 (*N* ≈ 350,000) (Engzell et al., 2021). Learning loss estimates were slightly larger for math than for reading (see also Kuhfeld et al., 2020), and the effect was larger in schools with a high proportion of students from non-academic families or migration backgrounds.

Sixth graders in Belgium (*N* ≥ 1287) experienced significant learning losses in math and Dutch language tests after the 9-week school lockdown in spring 2020, as compared with the previous cohort (Maldonado & De Witte, 2021). However, participation was voluntary for schools, and in 2020, the number of schools was reduced by more than 50% compared with previous years, which might have compromised the validity of comparison between the cohorts. Outside of Europe, the inter-individual comparison of the fourth graders in US public school samples of fall 2019 and fall 2020 showed small learning loss in reading but a more pronounced loss in math compared with previous years, although this was not related to family SES (Kuhfeld et al., 2020). In Canada, the study by Georgiou (2021) showed that the reading performance in Grades 2 and 3 in eight schools was lower in September 2020 than the average score from the previous 3 years.

However, there have been a few studies that reported a positive effect after the first wave of lockdown, especially in mathematics (Meeter, 2021; Spitzer & Musslick, 2021). For example, Spitzer and Musslick (2021) showed that students’ performance in math at K-12 (*N* = 2500) increased during the lockdown in Germany. This positive result might have been connected to the curriculum-based online learning software for mathematics used in these schools, which may have prevented learning loss in math. Also, in the study by Meeter (2021), students from Grades 2 to 6 (*N* = 53,656) in the Netherlands who used adaptive practice software for mathematics showed faster progress in their math learning during the lockdown compared with similar students in the preceding year. It might be that in this case, adaptive practice software mitigated the negative effects of school closures, at least on math learning and with low-achieving students. Also, in China (Clark et al., 2021), online education for ninth graders during the COVID-19 lockdown improved student academic results in Chinese and math. Notably, this result was associated with the quality of teaching: higher-quality online lessons were associated with higher exam scores, and especially the low achievers benefited from teaching quality. Moreover, Georgiou (2021) reported differences in the learning losses in English depending on grade levels in Canada indicating that children in Grades 2 and 3 were at greater risk of performing below grade level in reading compared to children in other grade levels.

### The role of gender

Gender differences in academic skills have been reported across school years (e.g., Manu et al., 2021; Vasilyeva et al., 2021). Number of studies have shown that girls perform better in reading than boys (e.g., Clinton et al., 2014; Mullis et al., 2017; Reilly et al., 2019; Torppa et al., 2018; Voyer & Voyer, 2014), whereas boys perform better in math (e.g., Lopez-Agudo & Ropero-Garcia, 2020; Stoet & Geary, 2013). Further, reading difficulties are more common among boys than girls (see e.g., Quinn & Wagner, 2013). The literature is mixed, however, as not all studies have found gender differences for example in the reading difficulty prevalence (e.g., Jimenez et al., 2011; Moll et al., 2014) nor in the average reading skill levels (e.g., McGeown et al., 2012; McIntosh et al., 2013). In Finland, gender difference appears to be particularly prominent (OECD, 2019) and present already in Kindergarten and elementary school (Manu et al., 2021). Therefore, we will study whether there were any gender-related differences at Grade 4 in the pre-COVID and the COVID sample.

### The role of the home environment

The studies described above have shown that family background and socio-economic variables of parents have affected student learning during pandemics. In sum, students’ learning loss was related to low education, low SES, and immigrant background of family (e.g., Engzell et al., 2021; Maldonado & De Witte, 2021; Schult et al., 2022). This result might be linked with the resources in the home environment that possibly help or hamper students’ learning, which takes place totally at home. Parents who are struggling with their own well-being or financial uncertainty, their remote work, or who speak a home language different from the child’s language of teaching might have limited resources to support their child’s distance learning at home (Van Bakel et al., 2022).

Parental assistance with homework has been the most typical parental involvement in their child’s schooling. Pomerantz and Eaton (2001) have differentiated two forms of parental control in homework situations: monitoring, which is typically defined as checking children’s homework, and helping, which demonstrates teaching or guiding a child in completing homework. However, previous research has shown mixed findings concerning the relation between parental homework assistance and students’ academic skills, partly due to the effect of students’ skill level, age of the child, or family SES. These included an increasing effect (Dumont et al., 2012; Patall et al., 2008), a decreasing effect (Cooper et al., 2000; Hill & Tyson, 2009; Silinskas et al., 2013), or no association (Cooper et al., 2000). Also, it has been discussed that different types of parental homework involvement and the child’s skill level might have different associations with children’s skill development (e.g., Patall et al., 2008; Silinskas et al., 2015), which partly explains the contradictory findings. For example, in a Finnish sample, Silinskas et al. (2010) showed that the poorer reading and math skills children had at the beginning of Grade 1, the more monitoring and help parents reported later on, but this had a negative association with their reading and math skills development. This result indicates that a child struggling with the homework will activate parents’ teaching, but that does not necessarily address the child’s learning difficulties.

However, total closure of schools during the first wave of the COVID-19 pandemic was a very different situation for children and parents compared with normal schooling with respect to homework. It is possible that the impact of home support is intensified for children in such situations. For example, Sonnenschein et al. (2021) showed that children increased digital activities at home significantly during COVID-19 and there was correlation between digital usage and home literacy activities. However, to our knowledge there has not yet been any research on the possible effect of parents’ involvement on a child’s learning outcomes during a pandemic. Therefore, we chose to study whether parental education and maternal homework involvement predicted children’s academic skills development during the pandemic and compared the effects in the COVID sample and the pre-COVID sample to better understand if the association was differential in the pandemic situation.

### The role of task avoidance

It is recognized that along with the environment, students’ individual behavior in learning situations has an impact on their performance (Grimm et al., 2010). Some students apply task-focused strategies showing persistence and engagement in challenging situations, while others engage in task-avoidant behavior showing resistance to challenging situations, avoiding difficult tasks, and withdrawing in the face of failure (Turner et al., 2002). Task avoidance refers to maladaptive behaviors that students display in response to challenges in academic tasks (Aunola et al., 2002).

Task avoidance has been shown to have a negative association with children’s reading (Aunola et al., 2002; Zhang et al., 2011) and math (Aunola et al., 2003; Zhang et al., 2011) performance. However, the findings are mixed and Georgiou et al. (2010) and Hirvonen et al. (2010) reported non-significant effects of task avoidance on reading fluency. In remote teaching, teachers cannot support task-focused behavior as efficiently as in a classroom situation. Students who tend to react to difficulties in task performance by avoidance may be at a particular risk for learning loss, as teacher or peer support may not be as quickly or easily at hand in online learning solutions as in a classroom. During the lockdown, students were reported to have spent considerably less time on learning activities at home (Grewenig et al., 2021; Huber & Helm, 2020). However, there are no studies showing whether task avoidance behavior predicts learning during school closure. In the present study, we investigated whether the association between task avoidance and academic skills was similar in the COVID sample and in the pre-COVID sample to better understand its role in the potential learning losses during the pandemic.

### The present study

The current study set out to provide further insights into the potential differences in academic skills between the COVID sample and the pre-COVID sample in Finland, extending the international findings discussed above. The current study investigated the extent to which children’s reading and math skills development differed between the COVID sample and the pre-COVID sample. The more specific research questions were as follows:

1. How do reading and math skill development trajectories across Grades 1, 2, and 4 differ between the COVID-sample and the pre-COVID sample?
2. Do gender, parental education, maternal homework involvement and child’s task-avoidant behavior predict children’s academic skills at Grade 4 differently in the pre-COVID sample than in the COVID sample when accounting for previous skill level?

## Method

### Participants and procedures

***Pre-COVID Sample.*** The sample was part of a large-scale longitudinal First Steps study (Lerkkanen et al., 2006–2016) where children (*N* = 2000) were followed from kindergarten to Grade 9 in four municipalities: one in an urban area, one in a rural area, and two in semi-rural areas in central, western, and eastern Finland, respectively. The study was reviewed and approved by the ethical committee of the university in 2006. Parents gave written consent for their own and their child’s participation in the study. Parents’ education was representative of the general Finnish population (Statistics Finland, 2010): 4.2% of the parents had no vocational education or only short vocational courses, 23.6% had a vocational school degree, 22.5% had a college-level degree, 13.2% had a polytechnic degree, 26.1% had a university degree, 5.1% had a licentiate or doctor’s degree, and 5.1% of parents did not report their education. The sample was also highly homogeneous in its ethnic, cultural, and language background (e.g., Finnish-speaking schools and students), which was characteristic of a school population outside the metropolitan region at that time. The pre-COVID sample was assessed in 2008–2011 following the same children at Grade 1 (*n* = 2056, 47.9% girls), Grade 2 (*n* = 2008, 47.9% girls), and Grade 4 (*n* = 1954, 47.5% girls). Children were tested in their academic skills, mothers filled out questionnaires, and teachers rated children’s task-avoidant behavior each spring. The subsample (*n* = 378 students; 48% girls) was randomly selected from the larger sample to ensure that the clearly larger sample size in the pre-COVID would not affect the results. We tested the randomly selected sample to ensure that it did not differ from the larger sample in terms of study variables (Appendix A). Children in the random sample showed less task avoidance and had higher arithmetic reasoning skills in Grade 2 than children in the full sample. In the full sample, mothers provided more help in Grade 3 than in the random sample. The COVID sample was compared with the subsample in the further analyses.

***COVID Sample.*** The data of the COVID sample were taken from a larger longitudinal study of Teacher and Student Stress and Interaction in Classroom (TESSI; Lerkkanen & Pakarinen, 2016–2022) conducted in Central Finland that examined the well-being of teachers and students and the quality of teacher–student interactions in Finnish classrooms. The research project received ethical approval from the university’s ethics committee in 2016. Parents gave written consent for their own and their child’s participation in the study. Parents’ education was representative of the general Finnish population: 0.4% of the parents had no vocational education or only short courses, 17.8% had a vocational school degree, 4.2% had a college-level degree, 17.3% had a polytechnic degree, 20.4% had a university degree, 4.8% had a licentiate or doctor’s degree, and 35% of parents did not report their education. The sample was also highly homogeneous in its ethnic, cultural, and language background. The COVID sample was composed of 877 (49.6% girls) Grade 1 students from 54 classrooms, 710 (50.7% girls) Grade 2 students from 50 classrooms, and 459 (54.9% girls) Grade 4 students from 38 classrooms. Children were tested in their academic skills, mothers filled out questionnaires, and teachers rated children’s task-avoidant behavior each spring. It should be noted, however, that in Grade 4, children’s reading fluency and reading comprehension were assessed at the end of the fall term (see Table 1). The children of this sample were in Grade 3 when COVID-19 closed the schools for 8 weeks in spring 2020. The schools were open again during the last 2.5 weeks before the children went on summer break for 10 weeks at the beginning of June. There was attrition in the sample due to the COVID-19 and not all the teachers and classrooms continued in the follow-up in Grade 4. We used Little’s MCAR test to test whether the data were missing completely at random. The results (χ ^2^(429) = 489.409, *p* < 0.05) indicated that the data were not missing completely at random. We then tested the differences between the follow-up sample (*n* = 198) and the larger COVID-sample (Appendix B). Children in the follow-up sample had higher reading comprehension in Grade 1, higher reading fluency in Grade 4, and higher arithmetic fluency in Grades 1–4. In addition, parental level of education was higher in the follow-up sample.

**Table 1:** Pre-COVID and COVID sample assessments and assessment time points

Assessment Time Point		Pre-COVID Sample				COVID Sample				
		1st Grade	2nd Grade	3rd Grade	4th Grade	1st grade	2nd grade	3rd grade	4th grade	
		March–April 2008	March–April 2009	March–April 2010	March–April 2011	February-May 2018	February-May 2019	March–May 2020	November 2020-February 2021	March–April 2021
	Range									
*Reading skills*										
Reading Fluency	0–80	X	X		X	X	X		X	
Reading Comprehension	0–12	X	X		X	X	X		X	
*Math skills*										
Arithmetic Fluency	0–28	X	X		X	X	X			X
Arithmetic Reasoning	0–30		X		X		X			X
*Task-avoidance*										
Task-Avoidant Behavior	1–5		X				X			
*Maternal Homework Involvement*										
Maternal Help	1–5		X	X			X	X		
Monitoring	1–5		X	X			X	X		
Autonomy Support	1–5		X	X			X	X		
*Parental Education*	1–7		X				X			

### Measures

The assessment points and measures for each of the assessment points are presented in Table 1. The Cronbach’s alpha reliabilities of the study variables in both samples are listed in Table 2.

**Table 2:** Descriptive Statistics and Pre-COVID and COVID Sample Comparisons of the Investigated Variables

	Pre-COVID sample				COVID-19 sample					
	*N*	*M*	*SD*	*α*	*N*	*M*	*SD*	*α*	*t*	*d*
Parental Education G1^1^	355	4.47	1.43		144	5.18	1.23		−5.244***	0.53
Gender^2^	377	1.52	0.50		198	1.46	0.50		1.374	0.12
Age G1^3^	377	7.75	0.29		198	7.75	0.36		−0.233	0
Age G2^3^	361	8.75	0.29		198	8.75	0.36		−0.208	0
Age G4^3^ (reading)	346	10.75	0.29		130	10.44	0.35		9.706***	0.96
Age G4^3^ (math)	346	10.75	0.29		177	10.74	0.34		0.286	0.03
*Reading skills*										
Reading Fluency G1	377	18.17	9.19	0.95	198	15.92	8.23	0.95	2.886**	0.26
Reading Fluency G2	365	24.39	7.59	0.97	198	21.15	7.70	0.92	4.808***	0.42
Reading Fluency G4	346	36.58	9.12	0.86	130	31.30	8.44	0.94	5.688***	0.60
Reading Comprehension G1	374	1.44	5.00	0.78	185	2.17	4.55	0.84	−.543	0.15
Reading Comprehension G2	356	5.14	5.26	0.82	198	5.18	5.48	0.78	−0.070	0.01
Reading Comprehension G4	346	4.45	4.93	0.82	128	2.80	4.64	0.67	3.292**	0.34
*Math skills*										
Arithmetic Fluency G1	377	10.56	4.24	0.85	198	10.77	4.73	0.86	−1.675	0.05
Arithmetic Fluency G2	364	16.29	4.92	0.85	198	16.54	5.99	0.91	−0.491	0.05
Arithmetic Fluency G4	346	17.05	4.10	0.89	185	17.59	4.46	0.94	−1.418	0.13
Arithmetic Reasoning G2	364	3.52	7.76	0.78	197	2.92	9.63	0.81	0.750	0.07
Arithmetic Reasoning G4	346	6.99	7.81	0.85	186	7.10	8.24	0.77	−0.154	0.02
*Task-Avoidance*										
Task-Avoidance G2	317	2.45	1.04	0.92	173	2.32	1.20	0.95	1.181	0.12
*Homework Iivolvement*										
Maternal Help G2	288	2.92	0.68	0.88	106	3.09	1.03	0.91	−1.569	0.19
Monitoring G2	289	3.93	0.83	0.85	106	4.19	0.86	0.86	−2.695**	0.31
Autonomy Support G2	288	3.85	0.83	0.82	106	3.79	0.86	0.81	0.674	0.07
Maternal Help G3	276	2.75	0.65	0.73	77	2.94	0.94	0.92	−1.718	0.24
Monitoring G3	275	3.54	0.86	0.85	77	3.90	0.87	0.85	−3.291**	0.42
Autonomy Support G3	275	3.80	0.84	0.85	77	3.87	0.93	0.83	−0.573	0.08

*Note*. G1 = Grade 1, G2 = Grade 2, G3 = Grade 3, G4 = Grade 4; ^1^Parental education, highest in the family; ^2^1 = girl, 2 = boy; ^3^age in years at the assessment

***Reading fluency.*** The word reading fluency task was an 80-item subtest of the nationally normed reading test battery (ALLU; Lindeman, 2000). Parallel versions of the test (A and B) were used every other year in Grades 1–4. Each item comprised a picture and a set of four phonologically similar words. The children were asked to silently read the words and decide which one semantically matched the picture. All the words and pictures in the task were simple and frequently used, and thus were familiar to young children. The score was calculated as the number of correct answers achieved within 2 min. The score reflected both word-reading speed and accuracy. In the analyses, we used a score of correct minus incorrect items.

***Reading comprehension.*** To assess reading comprehension in Grades 1–4, a group administered subtest of a nationally normed reading test battery was used (ALLU; Lindeman, 2000). The children were asked to read a short factual text and answer 11 multiple-choice questions and one question in which they had to arrange five statements in the correct sequence based on the information gathered from the text. The length of the text was five paragraphs with 124 words in total in Grade 1; five paragraphs with 114 words in Grade 2; and four paragraphs with 263 words in Grade 4. For each correct answer, one point was given (max = 12). The children could work at their own pace for a maximum of 45 min. In the analyses, we used a score of correct minus incorrect items.

***Arithmetic fluency***. Arithmetic fluency was assessed with a group-administered subtest of the arithmetic test (Aunola & Räsänen, 2007). At each time point from Grade 1 to Grade 2, the initial form containing 14 addition (e.g., 3 + 2 = __, 3 + 6 + 4 = __) and 14 subtraction (e.g., 6 − 1 = __, 20 − 4 − 3 = __) items were used. In Grade 4, six new and more difficult items of addition, subtraction, multiplication (e.g., 12 × 28 = __), division (e.g., 240 ÷ 80 = __), or mixed mode calculation (e.g., 40 ÷ 8 – 3 = __) were developed to replace the six easiest items (e.g., 4 – 1 = __, 2 + 1 = __) to match the fourth grade curriculum. Performance on this test depended on both speed and accuracy, and allowed for the assessment of the automatization of basic mathematical computations. The sum score was based on the number of correct answers given within 3 min.

***Arithmetic reasoning***. Arithmetic reasoning was assessed with the Arithmetic Reasoning Test (Räsänen, 2000) in Grades 2 and 4. The children were asked to continue a series of three numbers by adding a fourth number that would fit the series. They were first given a series of three numbers (e.g., 3, 5, 7). They were then shown four additional numbers, only one of which was correct to continue the given series. Finally, the children were asked to circle the number that would best fit as the fourth number. After the practice trials, the child started to complete 30 series of test trials with a time limit of 10 min. The test trials ranged in difficulty and involved rules ranging from one (e.g., 2, 4, 8) to two (e.g., 15, 7, 3) basic arithmetic functions (+ , –, × , ÷). The test involved complex and multistep problem-solving (analyzing the relation between the three numbers given, recognizing the arithmetic rule using inductive reasoning, and applying the arithmetic rule to predict the value of the fourth number). One point was given for each correct answer. In the analyses, we used a score of correct minus incorrect items.

***Parental education.*** Mothers were asked to indicate their own and their spouse’s educational level on a seven-point scale (1 = no vocational education, 2 = vocational courses, 3 = vocational school degree, 4 = vocational college degree, 5 = polytechnic degree or bachelor’s degree, 6 = master’s degree, and 7 = licentiate or doctoral degree). The highest vocational education in the family was used in further analyses.

***Task-avoidant behavior.*** Teachers rated children’s task-avoidant behaviors for Grade 2 using the Behavioral Strategy Rating Scale (Aunola et al., 2000; for validity, see Zhang et al., 2011), which consisted of five items on a 5-point scale (1 = never, 5 = always). They were asked to consider how a certain child typically behaved in different situations in school and then to rate their behavior using five statements (When facing difficulties, does the student tend to find something else to do instead of focusing on the task at hand? Does the student actively try to solve even the most difficult tasks (reversed)? Does it seem that the pupil easily gives up the task at hand? Does the student show persistence when working with the tasks? (reversed), and When problems occur with a task, does the student turn their attention to other things?). A mean score of five items were used in the analyses.

***Maternal homework involvement.*** Questions measuring the quantity of maternal homework involvement (i.e., monitoring and help) were adapted from Pomerantz and Eaton (2001) and Pomerantz and Ruble (1998; see also Silinskas et al., 2015, for validity in a Finnish sample) in Grades 2 and 3. Monitoring was measured with three items (Do you check your child’s homework? Do you make sure that your child has done his/her homework? Do you check your child’s homework together with your child?) using a 5-point scale (1 = never, 5 = always). Help was measured with four items (Do you instruct your child in his/her homework? Do you help or guide your child in his/her homework? Do you help your child in his/her homework related to reading? Do you help or guide your child in his/her homework related to mathematics?), which were rated with a 5-point scale (1 = never, 5 = always). Autonomy support was measured with three items on a 5-point scale (1 = never, 5 = always): Do you know that the child remembers to do their home assignments? Do you trust that the child takes care of their home assignments by themselves?, and Do you have to force your child to do the home assignments? (reversed). A mean score of items belonging to each domain was used in the analyses.

### Analysis strategy

The data were analyzed with IBM SPSS Statistics 27 (IBM, Armonk, NY, USA) and Mplus statistical package version 8.7 (Muthén & Muthén, 1998–2017). The repeated ANOVAs were conducted with SPSS and path models were determined with a multigroup analysis using Mplus. Due to slight skewness in some of the variables, the model parameters were estimated using the MLR estimator. Missing data were accounted for with FIML estimation in Mplus. Finally, the path estimates and correlations were compared between the COVID sample and the pre-COVID sample to see whether there were sample differences in the model estimates, which would then suggest that COVID moderated the associations. In the group comparison process, all path estimates, correlation, and error covariance estimates were set as equal, and the fit of the model was compared using the Satorra-Bentler scaled chi-square difference test with the baseline model, where all estimates were allowed to be freely estimated. If the test suggested that there was a group difference in the model, all model estimates were compared one by one.

## Results

The descriptive statistics and group comparisons of the study variables for the samples are reported in Table 2. There were no statistically significant differences between the samples in respect to the children’s gender or age in Grades 1–2 or at the time of the Grade 4 math assessment. Children in the COVID sample were, however, younger at the time of Grade 4 reading assessment compared to the pre-COVID sample. In addition, parental education was higher in the COVID sample. The reading skills were, on average, lower in the COVID sample than in the pre-COVID sample, except for Grade 1 and 2 reading comprehension. The effect sizes increased over time and showed small differences between the samples in reading fluency (*d* = 0.26) in Grade 1 but considerably larger differences in Grade 2 reading fluency (*d* = 0.42) and in Grade 4 for both reading comprehension (*d* = 0.34) and reading fluency (*d* = 0.60). In arithmetic fluency and reasoning, the pre-COVID sample did not significantly differ from the COVID sample. Mothers reported providing more monitoring of their children’s homework in the COVID sample in Grades 2 and 3 compared with the pre-COVID sample.

To examine sample differences in the development of reading fluency, reading comprehension, arithmetic fluency, and arithmetic reasoning across Grades 1 to 4, repeated measures ANOVAs were conducted (with the main effects of time and sample, and time × sample interaction). The analyses revealed a significant time × sample interaction in reading fluency, *F*(2, 421) = 9.707; *p* < 0.001; η^2^ = 0.023, and reading comprehension, *F*(2, 421) = 8.675; *p* < 0.001; η^2^ = 0.021, but not in arithmetic fluency or arithmetic reasoning. The development of reading fluency was slower in the COVID sample than in the pre-COVID sample across Grades 1 to 4, whereas the development of reading comprehension was similar in the samples between Grades 1 and 2 but slower in the COVID sample than in the pre-COVID sample between Grades 2 and 4, *F*(1, 398) = 11.372, *p* = 0.01, η^2^ = 0.028.

Next, we tested for gender interactions (gender × sample, gender × time, and gender × time × sample interactions). The analyses revealed a significant gender × sample interaction in reading fluency, *F*(1, 419) = 10.711; *p* < 0.01; η^2^ = 0.025, indicating that girls in the COVID-sample had slower development of reading fluency compared to boys while there were no gender differences in the development of reading fluency in the pre-COVID sample. There was also a significant gender × sample interaction in arithmetic fluency, *F*(1, 437) = 6.047; *p* < 0.05; η^2^ = 0.014, indicating that girls in the COVID-sample had slower arithmetic fluency development compared with boys. In addition, boys in the COVID-sample had higher arithmetic fluency in Grades 1 and 4 compared to boys in the pre-COVID sample.

Second, we investigated the roles of gender, parental education, task-avoidant behavior, and maternal homework involvement in reading and arithmetic development and whether their role was different in the COVID sample than in the pre-COVID sample. Table 3 reports the Pearson correlation coefficients between the variables in these two samples. Multiple significant correlations were identified between the constructs in both samples. In the models examining whether gender, parental education, task-avoidant behavior, and maternal homework involvement predicted reading and arithmetic skill development, we controlled for Grade 2 skill (autoregressor) to focus on the predictors of change from Grade 2 to 4. For the COVID sample, this time included the start of the pandemic and the remote teaching period. We specified separate models for reading fluency, reading comprehension, arithmetic fluency, and arithmetic reasoning. In the models, children’s age at the Grade 4 assessment was controlled for.

**Table 3:** Correlations between the Study Variables. COVID-sample above the diagonal and pre-COVID sample below the diagonal

	1	2	3	4	5	6	7	8	9	10	11	12	13	14	15	16
1.Reading Fluency G1	1	0.33^a^	0.46^a^	0.70^a^	0.35^a^	0.50^a^	0.21^b^	0.54^a^	0.43^a^	0.48^a^	0.45^a^	−0.23^b^	−0.37^a^	−0.36^a^	−0.11	−0.12
2. Reading Comprehension G1	0.43^a^	1	0.23^b^	0.37^a^	0.44^a^	0.33^a^	0.34^a^	0.25^c^	0.60^a^	0.35^a^	0.47^a^	−0.21^b^	−0.36^a^	−0.27^c^	−0.01	−0.19
3. Arithmetic Fluency G1	0.47^a^	0.30^a^	1	0.42^a^	0.25^a^	0.73^a^	0.41^a^	0.46^a^	0.29^a^	0.70^a^	0.52^a^	−0.33^a^	−0.36^a^	−0.35^b^	−0.25^c^	−0.25^c^
4. Reading Fluency G2	0.69^a^	0.39^a^	0.40^a^	1	0.38^a^	0.52^a^	0.23^a^	0.60^a^	0.45^a^	0.43^a^	0.33^a^	−0.24^a^	−0.32^a^	−0.24^a^	−0.12^c^	−0.05
5. Reading Comprehension G2	0.40^a^	0.54^a^	0.31^a^	0.42^a^	1	0.34^a^	0.43^a^	0.33^a^	0.57^a^	0.39^a^	0.43^a^	−0.38^a^	−0.30^a^	−0.30^a^	−0.10	−0.15^c^
6. Arithmetic Fluency G2	0.40^a^	0.28^a^	0.73^a^	0.39^a^	0.28^a^	1	0.42^a^	0.51^a^	0.42^a^	0.76^a^	0.50^a^	−0.34^a^	−0.34^a^	−0.36^a^	−0.15^b^	−0.17^c^
7. Arithmetic Reasoning G2	0.26^a^	0.35^a^	0.42^a^	0.22^a^	0.41^a^	0.38^a^	1	0.12	0.49^a^	0.47^a^	0.56^a^	−0.23^a^	−0.28^a^	−0.31^a^	−0.18^a^	−0.25^a^
8. Reading Fluency G4	0.60^a^	0.35^a^	0.45^a^	0.65^a^	0.37^a^	0.44^a^	0.25^a^	1	0.27^b^	0.61^a^	0.35^a^	−0.31^b^	−0.24^c^	−0.20	−0.10	−0.12
9. Reading Comprehension G4	0.26^a^	0.44^a^	0.25^a^	0.31^a^	0.57^a^	0.26^a^	0.36^a^	0.25^a^	1	0.41^a^	0.47^a^	−0.23^c^	−0.22	−0.29	0.11	0.02
10. Arithmetic Fluency G4	0.41^a^	0.32^a^	0.64^a^	0.40^a^	0.33^a^	0.71^a^	0.34^a^	0.51^a^	0.28^a^	1	0.50^a^	−0.45^a^	−0.43^a^	−0.38^a^	−0.28^b^	−0.34^b^
11. Arithmetic Reasoning G4	0.28^a^	0.37^a^	0.44^a^	0.32^a^	0.32^a^	0.43^a^	0.46^a^	0.25^a^	0.39^a^	0.45^a^	1	−0.18^b^	−0.41^a^	−0.41^a^	−0.22^c^	−0.39^a^
12. Task Avoidance G2	−0.21^a^	−0.29^a^	−0.32^a^	−0.19^b^	−0.41^a^	−0.37^a^	−0.25^a^	−0.25^a^	−0.35^a^	−0.36^a^	−0.23^a^	1	0.26^a^	0.30^a^	0.11^c^	0.16^c^
13. Maternal Help G2	−0.37^a^	−0.30^a^	−0.37^a^	−0.34^a^	−0.39^a^	−0.36^a^	−0.29^a^	−0.40^a^	−0.32^a^	−0.39^a^	−0.31^a^	0.25^a^	1	0.63^a^	0.49^a^	0.45^a^
14. Maternal Help G3	−0.26^a^	−0.25^a^	−0.32^a^	−0.23^a^	−0.33^a^	−0.33^a^	−0.24^a^	−0.27^a^	−0.31^a^	−0.36^a^	−0.29^a^	0.15^c^	0.49^a^	1	0.34^a^	0.51^a^
15. Monitoring G2	−0.22^b^	−0.22^b^	−0.26^a^	−0.18^b^	−0.21^b^	−0.21^b^	−0.20^b^	−0.21^b^	−0.16^c^	−0.25^a^	−0.23^a^	0.12	0.46^a^	0.28^a^	1	0.71^a^
16. Monitoring G3	−0.21^b^	−0.16^b^	−0.26^a^	−0.16^b^	−0.22^a^	−0.25^a^	−0.21^a^	−0.17^b^	−0.17^b^	−0.25^a^	−0.22^a^	0.16^c^	0.38^a^	0.43^a^	0.74^a^	1
17. Autonomy Support G2	0.07	0.05	0.05	0.05	0.13^c^	−0.02	0.09	0.05	0.00	−0.00	0.05	−0.20^b^	−0.017	−0.13	0.02	0.04
18. Autonomy Support G3	0.08	0.07	0.07	0.08	0.15^c^	0.05	0.05	0.17^b^	0.16^b^	0.07	0.08	−0.24^a^	−0.05	−0.14^c^	−0.10	−0.07
19. Gender^1^	−0.10	−0.05	0.01	−0.05	−0.21^b^	0.00	−0.03	−0.11	−0.20^b^	0.05	0.12	0.29^a^	0.09	0.03	0.05	0.10
20. Parental Education^3^	0.08	0.17^b^	0.05	0.14^c^	0.23^a^	0.06	0.19^b^	0.14^c^	0.27^a^	0.13^c^	0.21^b^	−0.19^b^	−0.16^c^	−0.05	−0.08	−0.08

COVID-sample	1	2	3	4	5	6	7	8	9	10	11	12	13	14	15	16
17. Autonomy Support G2	0.13	0.14	0.17	0.05	0.19^a^	0.16^b^	0.13^c^	0.02	0.19	0.15	0.05	−0.35^a^	−0.09	−0.15^c^	−0.08	−0.11
18. Autonomy Support G3	0.17	0.05	0.05	0.10	0.20^b^	0.11	−0.01	0.07	−0.07	−0.02	−0.08	−0.32^a^	−0.03	−0.19^b^	0.08	0.01
19. Gender^1^	0.18^b^	−0.06	0.22^b^	0.07	−0.12^a^	0.15^a^	−0.01	0.15	−0.05	0.18^b^	0.16^c^	0.32^a^	0.05	−0.01	−0.02	0.02
20. Parental Education^3^	0.13	0.45^b^	0.12	0.13	0.39^a^	0.24^a^	0.31^a^	0.02	0.43^a^	0.29^a^	0.19^c^	−0.29^b^	−0.20^c^	−0.35^b^	−0.19	−0.29^b^

	17	18	19	20												
17. Autonomy Support G2	1	0.47^a^	−0.12^c^	0.19												
18. Autonomy Support G3	0.53^a^	1	−0.15^c^	−0.07												
19. Gender^1^	−0.11	−0.13^c^	1	−0.01												
20. Parental Education^3^	0.08	0.13^c^	−0.03	1												

*Note.* G1 = Grade 1, G2 = Grade 2, G3 = Grade 3, G4 = Grade 4; ^a^*p* < 0.001, ^b^*p* < 0.01, ^c^*p* < 0.05. Two-tailed testing of significance; ^1^girl = 1, boy = 2; ^3^1 = no vocational education, 7 = doctor or licentiate

To examine sample differences in the effects of the predictors of reading and math development, we applied a multigroup analysis. In the multigroup models, the equality of the path estimates and correlations were tested between the COVID sample and the pre-COVID sample. In the multigroup procedure, all the estimates of the models were first set as equal in the samples, and the model fit was compared with a chi-square difference test to a base model where all the estimates were freely estimated in the samples.

**Fig. 1:**
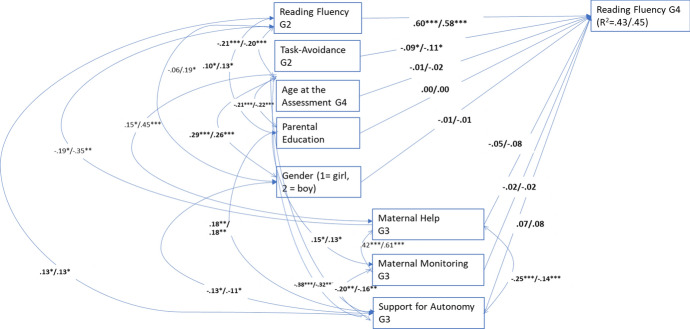
Multigroup model for reading fluency. Significant standardized path estimates, correlations and residual covariances. The first estimates are for pre-COVID sample (*n* = 344) and the latter for COVID-sample (*n* = 156). The estimates in bold were not significantly different. *p < 0.05; **p < 0.01; ***p < 0.001. G2 = Grade 2, G3 = Grade 3, G4 = Grade 4

The multigroup model of reading fluency with all estimates set equal did not fit the data well: χ^2^(44) = 82.631, *p* < 0.001, CFI = 0.84, RMSEA = 0.039 (90% CI = 0.039, 0.079), SRMR = 0.142. The chi-square difference test suggested that the estimates of the two groups were not equal, ∆χ^2^(28) = 54.842, *p* = 0.002. Therefore, the equality of each estimate was examined by setting each of the estimates as equal one by one and examining the significance of the model fit deterioration (chi-square difference testing) for each equality setting. The multigroup model comparisons of each estimate suggested that all paths and all but five correlations were equal in the groups. In the final model (see Fig. 1), all the paths and correlations that differed significantly between the groups were estimated freely, and the other estimates were fixed equal across the groups. The model fit the data well: χ^2^(39) = 53.346, *p* = 0.063, CFI = 0.94, RMSEA = 0.038 (90% CI = 0.000, 0.062), SRMR = 0.089.^.^The results indicated that the children who had less task-avoidant behavior in Grade 2 developed faster in reading fluency between Grades 2 and 4 in both samples.

**Fig. 2:**
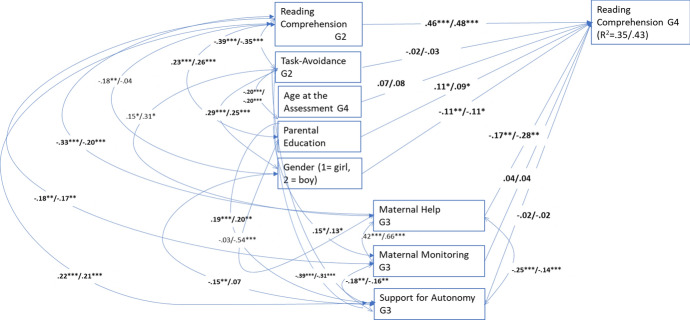
Multigroup model for reading comprehension. Significant standardized path estimates, correlations and residual covariances. The first estimates are for pre-COVID sample (*n* = 344) and the latter for COVID-sample (*n* = 156). The estimates in bold were not significantly different. *p < 0.05; **p < 0.01; ***p < 0.001. G2 = Grade 2, G3 = Grade 3, G4 = Grade 4

The multigroup model of reading comprehension with all estimates set as equal did not fit the data well: χ^2^(44) = 70.344, *p* = 0.01, CFI = 0.87, RMSEA = 0.049 (90% CI = 0.026, 0.070), SRMR = 0.134. The chi-square difference test, which compared this model with a model with all estimates freely estimated in the two samples, suggested that the models of the two samples were not equal, ∆χ^2^(28) = 44.074, *p* = 0.027. The multigroup model comparisons of each estimate suggested that all paths and all but five correlations were equal in the groups. In the final model (see Fig. 2), all the paths and correlations that differed significantly between the groups were estimated freely, and the other estimates were fixed equal across the groups. The final model fit the data well: χ^2^(39) = 42.920, *p* = 0.31, CFI = 0.98, RMSEA = 0.020 (90% CI = 0.000, 0.050), SRMR = 0.091. Higher parental education and less maternal help predicted better reading comprehension development in both samples. Gender negatively predicted reading comprehension development in both samples, with girls outperforming boys.

**Fig. 3:**
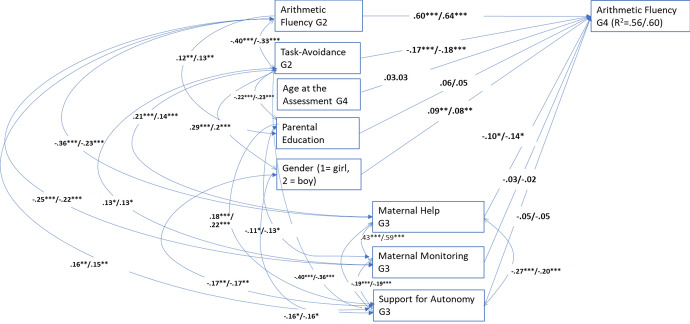
Multigroup model for arithmetic fluency. Significant standardized path estimates, correlations and residual covariances. The first estimates are for pre-COVID sample (*n* = 344) and the latter for COVID-sample (*n* = 198). The estimates in bold were not significantly different. *p < 0.05; **p < 0.01; ***p < 0.001. G2 = Grade 2, G3 = Grade 3, G4 = Grade 4

The multigroup model of arithmetic fluency with all estimates set as equal fit the data adequately: χ^2^(44) = 61.045, *p* = 0.05, CFI = 0.96, RMSEA = 0.037 (90% CI = 0.006, 0.055), and SRMR = 0.101. The chi-square difference test, which compared this model with a model with all estimates freely estimated in the two samples, suggested that the two samples were equal, ∆χ^2^(28) = 36.186, *p* = 0.14. The modification indices, however, suggested freeing one correlation to improve model fit. The fit of the final model (see Fig. 3) was: χ^2^(43) = 52.680, *p* = 0.15, CFI = 0.98, RMSEA = 0.028 (90% CI = 0.000, 0.053), and SRMR = 0.076. The children with less task-avoidant behavior developed faster in arithmetic fluency between Grades 2 and 4 in both samples. Gender positively predicted arithmetic fluency development in both samples, with boys outperforming girls. Less maternal help predicted better arithmetic fluency development significantly in both samples.

**Fig. 4:**
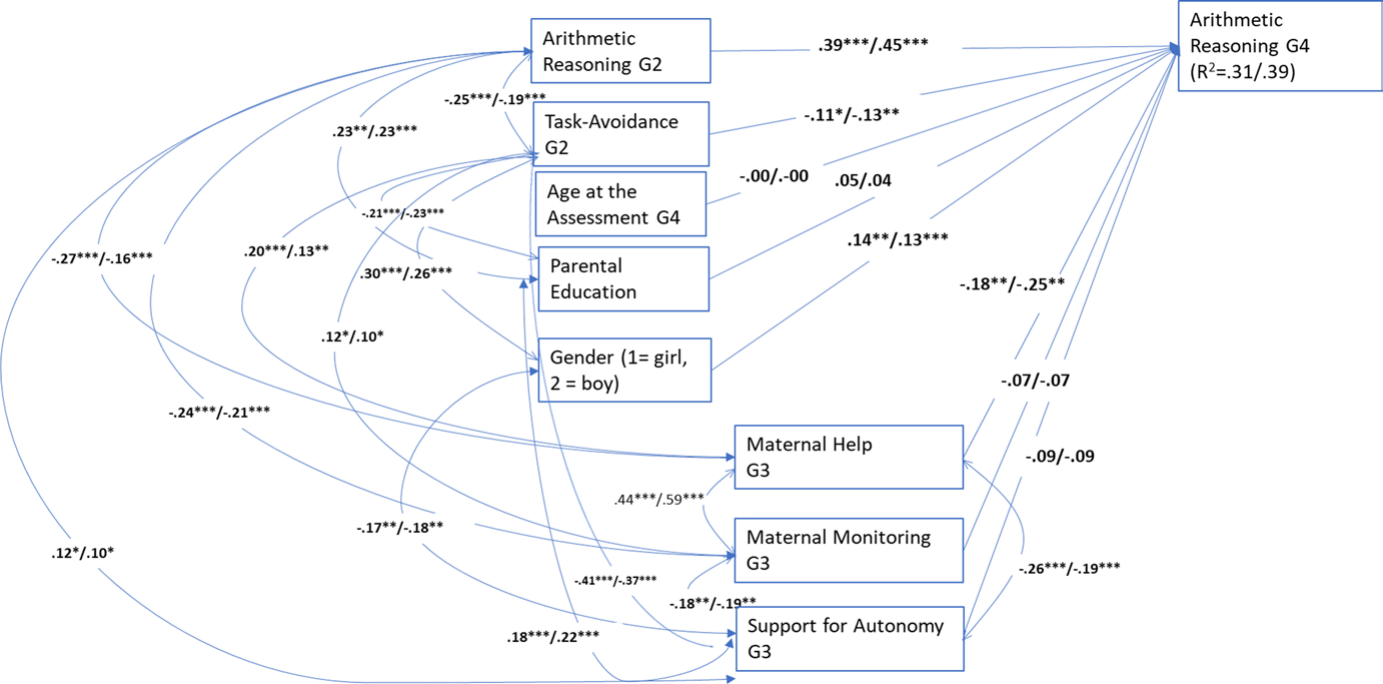
Multiplegroup model for arithmetic reasoning. Significant standardized path estimates, correlations and residual covariances. The first estimates are for pre-COVID sample (*n* = 344) and the latter for COVID-sample (*n* = 198). The estimates in bold were not significantly different. *p < 0.05; **p < 0.01; ***p < 0.001. G2 = Grade 2, G3 = Grade 3, G4 = Grade 4

The multigroup model of arithmetic reasoning with all estimates set as equal fit the data adequately: χ^2^(44) = 61.401, *p* = 0.04, CFI = 0.90, RMSEA = 0.037 (90% CI = 0.007, 0.058), and SRMR = 0.107. The chi-square difference test, which compared this model with a model with all estimates freely estimated in the two groups, suggested that the models of the two groups were equal: ∆χ^2^(28) = 39.377, *p* = 0.07. The modification indices, however, suggested freeing one correlation to improve model fit. The fit of the final model (see Fig. 4) was: χ^2^(43) = 54.579, *p* = 0.11, CFI = 0.93, RMSEA = 0.030 (90% CI = 0.000, 0.053), and SRMR = 0.081. The children with less task-avoidant behavior and less maternal help developed faster in arithmetic reasoning in both samples. Gender positively predicted arithmetic reasoning development, with boys outperforming girls in both samples.

## Discussion

The present study focused on possible learning losses in Finland during the COVID-19 pandemic. We compared children’s reading and math skills development from Grades 1 to 4 between a COVID sample and a pre-COVID sample. The children in the COVID sample were in Grade 3 when the pandemic started and the school 8-week closure took place. In addition, we examined whether gender, parental education, child’s task-avoidant behavior, and mother’s homework involvement predicted academic skill development and if the pandemic situation moderated the associations. The results suggest that the development of reading skills in particular may have been affected by school closure due to the COVID-19 pandemic. The developmental trajectories of the COVID sample in reading comprehension did not differ from the pre-COVID sample before the pandemic, but from Grade 2 to Grade 4, the development in reading comprehension of the COVID sample was slower. The gap also grew for reading fluency over time. Similar sample differences were not found for arithmetic fluency or arithmetic reasoning. Significant predictors of skill development from Grades 2 to 4 were found, and they were the same in the samples.

The results demonstrating learning losses in reading associated with COVID-19 and the related school closure are in line with most studies from other countries among primary school children (e.g., Clark et al., 2021; Engzell et al., 2021; Maldonado & De Witte, 2021; Schult et al., 2021). However, Georgiou (2021) and Kuhfeld et al. (2020) reported differences between grade levels reporting no learning losses in reading comprehension for Grade 3 onward. This might indicate that children in Grades 1 to 3 are still in the process of learning to read and need systematic instruction from their teachers while older, already independent readers do not rely as much on their teachers. In our samples, the most robust effect was found for reading comprehension. The effects on reading comprehension were probably due to less time spent on reading activities and less direct and exact instruction and feedback from teachers (Grewenig et al., 2021). This suggestion seems plausible also in our context, as in Grades 3 and 4 in Finland, when the COVID sample experienced the school closure, reading instruction focused heavily on reading comprehension. Reading comprehension has also been shown to be associated with the amount of reading (e.g., Torppa et al., 2020), and it may be that during remote schooling, children were reading less than during the pre-COVID times (see Sonnenschein et al., 2021). Reading fluency, on the other hand, has been found to be very stable from early grades onwards and less impacted by environmental effects (e.g., Torppa et al., 2020).

However, similar evidence for learning loss was not found in math development. This may reflect teachers’ stronger emphasis on mathematical tasks that students could practice at home on their own during the school closure. Math might also be easier to teach remotely and for parents to assist their children in primary school-level mathematics (Maldonado & De Witte, 2020). This finding is in contrast to several previous studies reporting learning loss in math (e.g., Engzell et al., 2021; Kuhfeld et al., 2020; Maldonado & De Witte, 2021; Schult et al., 2021), while few studies have reported similar and even positive effects on math (Meeter, 2021; Spitzer & Musslick, 2021). For example, Kuhfeld et al. (2020) showed serious learning losses in mathematics after COVID-19 school closure compared to typical school years among students from Grades 3 to 7 in the US sample. However, it is challenging to compare results on learning losses between countries because of differences in the reading and math tasks used in different studies. In addition, the educational systems and curricula differ, digital infrastructure as well as teachers’ and students’ digital skills differ, and each country made its own decisions concerning the period of school closure and teacher practices on distance learning. The shared experience is, however, that the rapid change to remote teaching in spring 2020 did not provide schools with adequate time to prepare teachers, students, and parents for distance learning.

The predictors of reading and arithmetic skills were the same across the samples. First, the stability estimates did not differ, suggesting that with or without COVID, Grade 2 skills predicted Grade 4 skills significantly. Gender differences were mostly similar in the two samples. It should be noted that girls in the COVID-sample had slower development of reading fluency compared to boys. In line with previous studies, girls performed better in reading comprehension (e.g., Mullis et al., 2017; Reilly et al., 2019), whereas boys performed better in arithmetic tasks (e.g., Lopez-Agudo & Ropero-Garcia, 2020; Stoet & Geary, 2013). Task avoidance also predicted reading fluency, arithmetic fluency, and arithmetic reasoning similarly in both samples. In line with previous studies (Aunola et al., 2002, 2003; Zhang et al., 2011), the less task-avoidant behavior the child was reported to show, the better their skills were. However, this finding is partly contradictory with a previous study by Hirvonen et al. (2010) which showed a non-significant relation between task avoidance and reading fluency in a Finnish sample. Finally, the effect of parental education on children’s reading fluency, arithmetic fluency, and arithmetic reasoning development was nonsignificant in both samples. These findings suggest that gender, parental education, and task avoidance were not specific risk factors during COVID times for reading fluency, arithmetic fluency, or arithmetic reasoning. As the effects were similar in both samples, the lack of task avoidance may also be conceptualized as a promotive factor that supports skill development, both in the presence or absence of risks (COVID). Protective factors, on the other hand, are particularly important in the presence of risks or in risky times, such as COVID (e.g., McGrath et al., 2020).

The finding of the effect of parental education on reading comprehension is in line with previous studies (e.g., Khanolainen et al., 2020; OECD, 2019). The effects of parents' education can be indirect through children’s access to literacy resources at home. More educated parents provide more frequent access to literacy resources and perhaps better-quality resources than less educated parents. Prior studies have suggested that highly educated mothers provide a better home literacy environment than parents with lower education (e.g., Hamilton et al., 2016; Khanolainen et al., 2020; Park, 2008). Sonnenschein et al. (2021) also reported that children increased not only digital activities but also the use of home literacy activities significantly during COVID-19.

The results showed that less maternal help at home predicted better reading comprehension development in both samples. It is plausible that the association reflects the greater needs children (see also Silinskas et al., 2013). At this stage of the school career, parents have received feedback from the school and were likely helping more the children who needed more help. The results also showed an increased difference in the amount of homework monitoring (*d* = 0.31 → *d* = 0.42) from Grade 2 to 3 in the COVID sample compared with the pre-COVID sample. This study thereby confirms that the total closure of schools during the first wave of the COVID-19 pandemic put parents in a very different situation with respect to supporting their child’s learning. Observing their child’s distance learning from a very different and more proximal perspective on a daily basis intensified the homework support for children during school closure.

### Limitations

There are a number of potential limitations to this study that must be taken into account. First, the comparisons of the results of the COVID sample from the years 2018–2021 to the pre-COVID sample from 2008 to 2011 may reflect not just effects of the COVID and related school closure but also the overall decline of reading and math competence of Finnish students (OECD, 2019). However, as the skill levels were comparable, except for reading fluency, in Grades 1 and 2, this should not be a major issue with the current findings. Second, the obvious variability of students’ distance learning practices at home and the quality of remote teaching made it difficult to show the causal effect of specific features or practices during the school closures on student achievement. Third, the percentage of parents who did not respond in the COVID sample was significantly different from that of the pre-COVID sample. The reason might be the pandemic situation in the families which was stressful also to the parents and affected their motivation to continue in the study. It is also possible that some of the Grade 2 variables predict children's reading or math indirectly through mothers' involvement in Grade 3. This should be investigated more thoroughly in future studies. Finally, the present study did not focus on low-achieving students or students with migration backgrounds per se, although previous studies have shown that school closure might have serious consequences, especially for their learning, and may increase the risk of dropping out of education. Thus, analysis of the longitudinal effects of school closure on the educational path of these risk groups are needed.

## Conclusions

The present study indicates that there was a drop particularly in the reading comprehension of Finnish fourth graders during the COVID-19 pandemic that led to 8 weeks of school closure in spring 2020 when students were in the third grade. These results complement previous findings that revealed learning loss in academic achievement because of pandemics. Based on the results, we suggest that policy recommendations for schools and teachers are needed and that teachers’ guidance for parents throughout the pandemic might have mitigated the effects of school closure on student achievement to some degree.

## Appendix A

**Table:** Descriptive statistics and pre-COVID and full pre-COVID sample comparisons of the investigated variables

	Pre-COVID sample (random sample)			Full Pre-COVID sample				
	*N*	*M*	*SD*	*N*	*M*	*SD*	*t*	*d*
Parental Education G1^1^	355	4.47	1.43	1479	4.48	1.48	0.111	0.01
Gender^2^	377	1.52	0.50	1679	1.52	0.50	0.065	0
Age G1^3^	377	7.75	0.29	1608	7.76	0.33	0.129	0.03
Age G2^3^	361	8.75	0.29	1364	8.75	0.33	0.133	0
Age G4^3^ (reading)	346	10.75	0.29	1503	10.75	0.32	0.160	0
Age G4^3^ (math)	346	10.75	0.29	1503	10.75	0.32	0.160	0
*Reading skills*								
Reading Fluency G1	377	18.17	9.19	1673	17.84	9.25	−0.625	0.05
Reading Fluency G2	365	24.39	7.59	1640	23.61	7.84	−1.728	0.10
Reading Fluency G4	346	36.58	9.12	1608	35.52	9.35	−1.914	0.11
Reading Comprehension G1	374	1.44	5.00	1660	1.14	4.78	−1.074	0.06
Reading Comprehension G2	356	5.14	5.26	1582	5.26	5.14	0.224	0.02
Reading Comprehension G4	346	4.45	4.93	1604	4.29	4.90	−0.571	0.03
*Math skills*								
Arithmetic Fluency G1	377	10.56	4.24	1673	10.50	4.10	−0.273	0.01
Arithmetic Fluency G2	364	16.29	4.92	1637	16.00	4.92	0.661	0.06
Arithmetic Fluency G4	346	17.05	4.10	1607	17.03	4.09	−0.087	0.00
Arithmetic Reasoning G2	364	3.52	7.76	1631	2.45	8.31	−2.235*	0.13
Arithmetic Reasoning G4	346	6.99	7.81	1605	6.14	7.76	−1.840	0.11
*Task-avoidance*								
Task-Avoidance G2	317	2.45	1.04	182	3.10	1.06	6.663***	0.62
*Homework Involvement*								
Maternal Help G2	288	2.92	0.68	1168	2.98	0.77	1.125	0.08
Monitoring G2	289	3.93	0.83	1171	3.96	0.83	0.469	0.04
Autonomy Support G2	288	3.85	0.83	1171	3.81	0.85	−0.652	0.05
Maternal Help G3	276	2.75	0.65	1083	2.86	0.69	2.487*	0.16
Monitoring G3	275	3.54	0.86	1083	3.64	0.81	1.846	0.12
Autonomy Support G3	275	3.80	0.84	1083	3.79	0.85	−0.223	0.01

*Note*. G1 = Grade 1, G2 = Grade 2, G3 = Grade 3, G4 = Grade 4; ^1^Parental education, highest in the family; ^2^1 = girl, 2 = boy; ^3^age in years at the assessment

## Appendix B

**Table:** Descriptive statistics and Follow-up COVID sample and Full COVID Sample Comparisons of the Investigated Variables

	COVID sample (Follow-up)			Full COVID sample				
	*N*	*M*	*SD*	*N*	*M*	*SD*	*t*	*d*
Parental education G1^1^	144	5.18	1.23	426	4.70	1.38	−3.701***	0.37
Gender^2^	198	1.46	0.50	679	1.52	0.50	1.420	0.12
Age G1^3^	198	7.75	0.36	673	7.75	0.34	−0.073	0
Age G2^3^	198	8.75	0.36	505	8.77	0.35	0.646	0.06
Age G4^3^ (reading)	130	10.44	0.35	115	10.46	0.33	0.566	0.06
Age G4^3^ (math)	177	10.74	0.34	187	10.80	0.32	1.600	0.18
*Reading skills*								
Reading Fluency G1	198	15.92	8.23	676	16.59	9.17	0.920	0.08
Reading Fluency G2	198	21.15	7.70	512	19.98	6.54	−1.899	0.16
Reading Fluency G4	130	31.30	8.44	113	28.30	7.09	−2.973**	0.38
Reading Comprehension G1	185	2.17	4.55	597	0.28	4.86	−4.674***	0.40
Reading Comprehension G2	198	5.18	5.48	506	4.62	5.02	−1.291	0.11
Reading Comprehension G4	128	2.80	4.64	115	2.48	4.90	−0.520	0.07
*Math skills*								
Arithmetic Fluency G1	198	10.77	4.73	679	9.47	4.19	−3.535***	0.29
Arithmetic Fluency G2	198	16.54	5.99	512	15.29	5.03	−2.792**	0.23
Arithmetic Fluency G4	185	17.59	4.46	259	16.45	3.85	−2.887**	0.27
Arithmetic Reasoning G2	197	2.92	9.63	511	2.11	9.00	−1.055	0.09
Arithmetic Reasoning G4	186	7.10	8.24	259	7.07	7.44	0.144	0.00
*Task-avoidance*								
Task-Avoidance G2	173	2.32	1.20	399	2.37	1.22	0.420	0.04
*Homework Iivolvement*								
Maternal Help G2	106	3.09	1.03	295	3.06	1.01	−0.242	0.03
Monitoring G2	106	4.19	0.86	295	4.04	0.90	−1.465	0.17
Autonomy Support G2	106	3.79	0.86	295	3.77	0.89	−0.145	0.02
Maternal Help G3	77	2.94	0.94	232	3.07	1.03	1.029	0.13
Monitoring G3	77	3.90	0.87	232	3.92	0.91	0.161	0.02
Autonomy Support G3	77	3.87	0.93	232	3.76	0.89	−0.919	0.12

*Note*. G1 = Grade 1, G2 = Grade 2, G3 = Grade 3, G4 = Grade 4; ^1^ Parental education, highest in the family; ^2^ 1 = girl, 2 = boy; ^3^ age in years at the assessment
